# Management of Cavernous Sinus Thrombosis Following Herpes Zoster Ophthalmicus

**DOI:** 10.7759/cureus.21036

**Published:** 2022-01-08

**Authors:** Yusuf Mehkri, Jeff A Valisno, Lorena Figueredo Rivas, Jairo Hernandez, Varun Jain, Aisha Elfasi, Justin De Prey, Calvin Hu, Bedirhan Tarhan, Ibrahim S Tuna, Hans H Shuhaiber

**Affiliations:** 1 Department of Neurosurgery, University of Florida College of Medicine, Gainesville, USA; 2 Department of Neurology, University of Florida College of Medicine, Gainesville, USA; 3 Department of Radiology, University of Florida College of Medicine, Gainesville, USA

**Keywords:** varicella-zoster virus, herpes zoster meningitis, herpes simplex ophthalmicus, herpes simplex, aseptic cavernous sinus thrombosis

## Abstract

Herpes zoster opthalmicus (HZO) is the reactivation of latent varicella zoster virus (VZV) within the ophthalmic branch of the trigeminal ganglion (V1). Common complications are postherpetic neuralgia and vasculopathy. Here, we report a rare case of a 47-year-old female presenting with HZO and aseptic cavernous sinus thrombosis (CST). Early screening for rare and deadly complications such as CST using CT cerebral venography (CTV) and magnetic resonance venography (MRV), as was done, is crucial to detection at earlier stages when intervention is most effective. Anticoagulation therapy was promptly started, and the patient’s symptoms continued to improve during the hospital stay.

## Introduction

Herpes zoster (HZ) is defined as the reactivation of latent varicella-zoster virus (VZV) in sensory ganglia. When the virus is reactivated, vesicular rash and pain can appear within a specific dermatomal distribution [1]. Viral reactivation is associated with impaired immunity with aging, trauma, and psychological stress. Herpes zoster ophthalmicus (HZO) is the reactivation of VZV in the ophthalmic branch (V1) of the trigeminal nerve (CN V) [2]. It is estimated that one million cases of HZ present annually in the United States, and HZO accounts for 10% to 20% of those cases [1,2]

HZO typically presents with a unilateral V1 dermatomal distribution of prodromal pain, which could be described as eye pressure, redness, or tearing. Many patients also experience prodromal fever, headache, and malaise, which could progress with a row of vesicular or pustular rash to the same area [3]. The presence of herpetic lesions around the tip of the nose is known as the Hutchinson sign, which is an index of nasociliary branch involvement of V1. The Hutchinson sign confers an increased risk for ocular involvement since the nasociliary branch innervates the tip of the nose, cornea, and other ocular structures [4]. Potential complications of HZO include conjunctivitis, blepharitis, keratitis, uveitis, secondary glaucoma, postherpetic neuralgia, and permanent vision changes or loss [5]. The prognosis of HZO is highly variable and dependent on many risk factors and how early treatment is initiated [3,4,5]. We present a case of a 47-year-old female displaying a classic presentation of HZO with a rare complication of cavernous sinus thrombosis.

## Case presentation

The patient is a 47-year-old female with a past medical history significant for untreated Hepatitis C and hypertension who presented to another hospital with a headache, progressively increasing eye pain and decreasing vision, stiff neck, and itchy facial rash. The other hospital was concerned for HZO and gave the patient IV acyclovir prior to transfer to our emergency department. On examination, the patient had a clear vesicular rash on the right forehead, nose, and cheek. She also had corneal injection with significant pain, periorbital edema limiting eye-opening, and reduced vision on the right side. In addition, the patient had a nonfocal neurological exam, full extraocular muscle movement, and no discharge from the eye.

**Figure 1:**
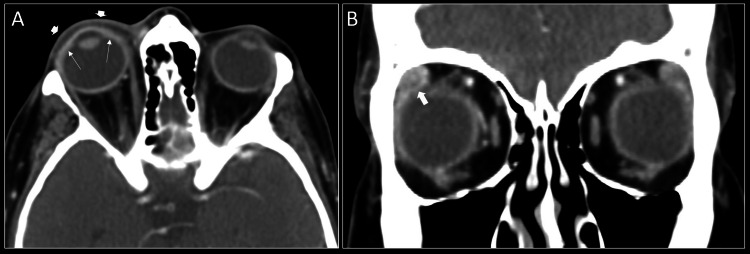
Contrast-enhanced CT orbits in axial (A) plane demonstrating asymmetric swelling and enhancement of the right preseptal periorbital soft tissues (arrowheads) and right cornea (arrow) compared to left, and coronal (B) plane showing asymmetric swelling and enhancement of the right lacrimal gland (thick arrow).

**Figure 2:**
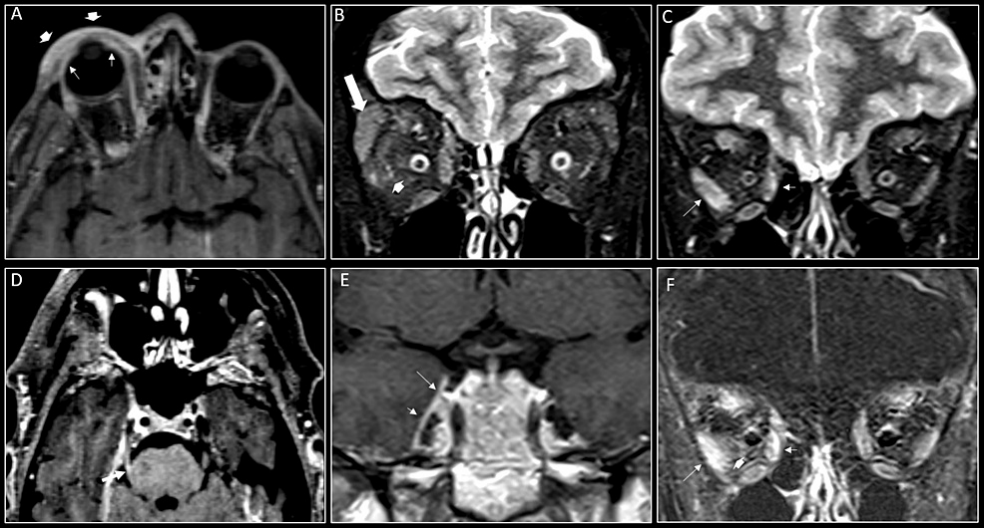
MRI of the cavernous sinuses were obtained including axial postcontrast 3DT1 space (A and D), coronal T2 fat saturated (B and C), coronal postcontrast T1 (E) and coronal postcontrast T1 subtracted from pre-contrast (F) images. There is asymmetric swelling and enhancement of the right pre-septal orbital soft tissues (arrowheads on A) and right cornea (arrow on A). There is asymmetric swelling and enhancement of the right intra-orbital fat (arrowhead on B and F) and right sided rectus muscles particularly involving the lateral rectus (long arrow on C and F) and medial rectus muscles (short arrow on C and F) as well as right lacrimal gland (thick arrow on B). There is evidence of intracranial extension with subtle asymmetric enhancement of the cisternal segment of the right trigeminal nerve (arrow on D) as well as asymmetric meningeal enhancement of the lateral wall of the right cavernous sinus (long arrow on E) and right Meckel’s cave (short arrow on E).

A contrast-enhanced CT scan of the orbits demonstrated asymmetric swelling and enhancement of the right preseptal soft tissues and right lacrimal gland and asymmetric thickening and enhancement of the right cornea (Figure 1). An MRI of the cavernous sinuses showed asymmetric swelling and enhancement of the right preseptal orbital soft tissue, right cornea as well as intraorbital fat, and right-sided rectus muscles particularly involving the lateral rectus and medial rectus muscles. There was evidence of intracranial extension with an asymmetric meningeal enhancement of the later wall of the right cavernous sinus and right Meckel’s cave. There was also subtle asymmetric enhancement of the cisternal segment of the right trigeminal nerve (Figure 2). The patient was admitted for management of HZO and possible herpes zoster meningitis (HZM). She was started on a 14-day course of IV acyclovir for HZO and broad-spectrum antibiotics (vancomycin and ceftriaxone) for empiric meningitis coverage.

**Figure 3:**
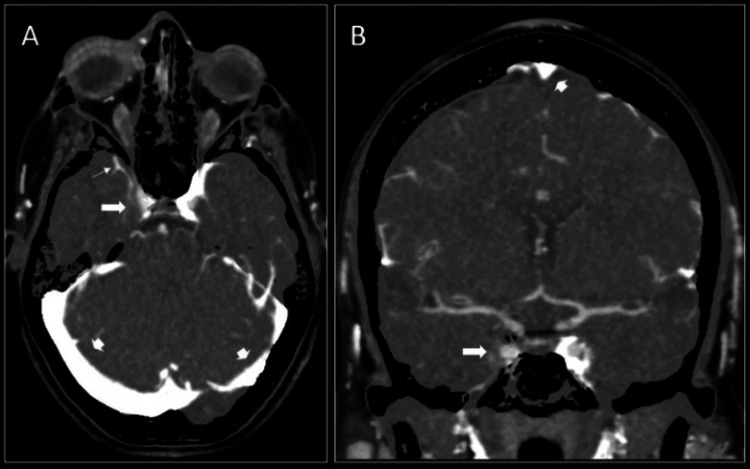
Subtracted axial (A) and coronal (B) maximum intensity projection imaging of the contrast-enhanced CTV demonstrates asymmetric filling defect and hypo-enhancement in the right cavernous sinus (thick arrow on A and B) and petrosal sinus (arrow on A) compared to left compatible with thrombosis. The remaining dural sinuses including transverse sinuses (arrowheads on A) and superior sagittal sinus (arrowhead on B) are patent. CTV: computerized tomography venography

Over the course of the day, the patient developed exquisite V1 distribution pain limiting eye movement, as well as meningeal signs such as worsening headache, photophobia, and positive Kernig’s and Brudzinski’s signs. Given clinical worsening, and inability to tolerate light, we were unable to photograph lesions during initial evaluations. Contrast-enhanced CT venography (CTV) of the head demonstrated asymmetric filling defect of the right cavernous sinus and right petrosal sinus compatible with cavernous sinus thrombosis (CST), whereas the remaining major dural sinuses were patent (Figure 3). The patient was therefore urgently started on full-intensity heparin gtt (15 units/kg/hr).

Lumbar puncture demonstrated normal opening pressure. CSF analysis showed: elevated protein (53), lymphocytes (96), red blood cells (32), white blood cells (32), and detection of the varicella-zoster virus (via PCR). Given the lack of concern for other sources of meningitis, empiric antibiotic coverage was discontinued. For long-term anticoagulation, she was later transitioned to warfarin 5mg/day. To treat herpetic neuralgia, she was started on pregabalin 100mg twice daily. For pain management, she was given oxycodone 5mg and ketorolac 15mg as needed.

**Figure 4:**
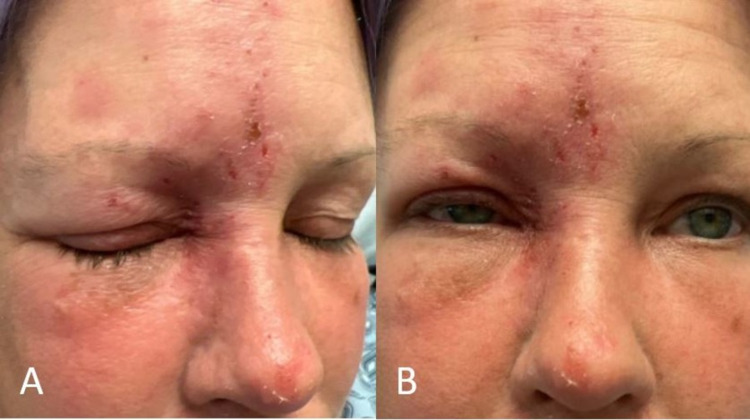
Appearance on day three of IV antiviral therapy.

On day three of IV Acyclovir therapy (day two in our hospital), the patient was noted to have new-onset infraorbital edema on the left eye, raising concern for dermatomal spread. To target the high degree of inflammation, the patient was started on a three-day treatment of IV pulse methylprednisolone (Solu-Medrol). Significant improvement was noted within the next 24 hours after steroid initiation, with a reduction in periorbital swelling allowing for an active eye-opening and full evaluation of extraocular movements (Figure 4A). Crusting of lesions was also noted; pain and conjunctival injection persisted (Figure 4B).

**Figure 5:**
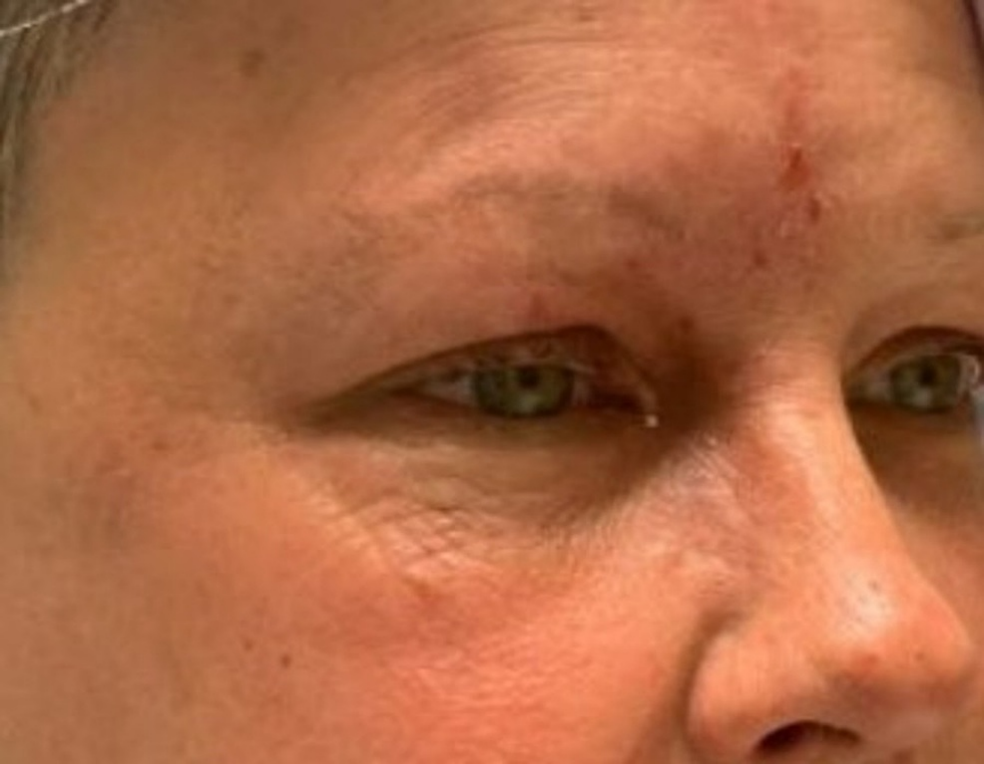
Appearance on day seven of admission.

Over the next few days, her condition further improved. Although her headaches and neck pain remained and required pain control, she appeared better and had significantly improved periorbital swelling (Figure 5).

## Discussion

VZV resides in sensory nerve cells during its latency period after the initial clinical presentation (chickenpox) [6]. Reactivation of latent virus in CN V1 results in HZO. An erythematous vesicular rash, often accompanied by prodromal pain, with dermatomal distribution about the V1 domain is a pathognomonic sign of this condition. Diagnosis is further confirmed through PCR testing of zoster rash swabs. VZV’s latent form is primarily suppressed by cell-mediated mechanisms [6], thereby age (with regards to cellular senescence), immunocompromised conditions (HIV, obesity, diabetes), cardiovascular disease, psychological stress, physical trauma, malignancies, and smoking are risk factors [7]. Females were also found to be at greater risk than males [7]. Our patient is positive for several risk factors including her female sex, BMI of 29.52, and history of type 2 diabetes, ovarian cyst, and myocardial infarction. Although immunization is available and recommended for adults greater than age 50, our patient is 47 years old and has therefore not been immunized.

Treatment by systemic antivirals (acyclovir, valacyclovir, famciclovir) is strongly supported by placebo control trials and was shown to reduce ocular complication (ie. vision loss associated with keratitis and uveitis) [8]. Our patient was started on IV acyclovir upon initial presentation to another hospital. Apart from ocular manifestations, the most common complication is postherpetic neuralgia affecting up to 20% of patients [9], but uncommon neurological complications such as encephalitis [10], meningitis [11], and, to a lesser extent, CST [12] have been reported. Here we report a case of a 47-year-old female who presented with HZO, early signs of CST, and zoster meningitis. Aside from one other report of cavernous sinus syndrome in a 62-year-old male in acute HZO [13] and a 44-year-old female with septic CST and HZO [14], we are the first to report a case of a middle-aged female with HZO, HZM, and CST.

CST is more commonly seen as a result of bacterial infection to the face, nose, soft palate, teeth, and ears, such as in a reported case of HZO masking septic CST in Korea [14]. Our patient presented afebrile with no other signs of bacterial infection. Although specific mechanisms for aseptic CST post-VZV infection are still undetermined, it is understood that local inflammation due to VZV infection promotes coagulation processes systemically and has been shown to result in deep vein thrombosis, pulmonary embolism, and cortical venous thrombosis [15]. In our case, the affected nerve, V1, passes through the cavernous sinus increasing the risk of local clots and providing the virus a route to infect adjacent nerves (CNIII, IV, VI) and the internal carotid artery leading to ophthalmoplegia and vasculopathy, a well-known complication. In addition to early signs of CST, our patient presented with pain associated with right extraocular muscle movement, sensations of sand in the right eye, and inability to open the right eye.

CST is a rare subtype of cerebral venous thrombosis (CVT), an uncommon but life-threatening cause of stroke [16]. Imaging modalities such as CTV and MRV are used to diagnose CST when there is sufficient clinical suspicion. In this setting, the American Heart Association claims that anticoagulation therapy is safe and effective, based on available data [16]. With strong clinical suspicion for a right cavernous sinus thrombosis based on CTV, our patient was started on 24-hour heparin replaced by warfarin with an international normalized ratio (INR) goal of 2.0-3.0.

In reports of CST, after primary varicella infection in adults, diagnosis with CST mostly occurred two to three weeks after onset of initial symptoms (i.e., skin rash), at which time patients usually presented with headache and/or seizure prompting a workup for potential CST [12]. Similarly, the case of a 62-year-old with HZO had onset of symptoms for CST 2.5 weeks after developing herpetic rash [13]. It is understood that earlier intervention leads to better outcomes. Our patient was diagnosed with CST within four days of prodromal pain and two days after onset of vesicular rash. Although CST was not well evaluated with MRV, early signs of CST were detected on CTV. This allowed our team to initiate comparatively early intervention via anticoagulant therapy, lowering the risk of complications associated with later interventions (cerebral edema and sudden cardiopulmonary arrest) [17].

With early suspicion for HZM, our patient was started on IV acyclovir within two days of initial symptom onset, in addition to vancomycin and ceftriaxone. A Finnish study on immunocompetent patients within one hospital estimated aseptic meningitis incidence to be at 7.6 per 100,000 patients, while approximately 8% of these patients’ meningitis was a consequence of VZV infection [11]. Presentation of HZO with CST and signs of HZM makes our case incredibly rare. Early intervention slowed disease progression and improved her hospital course. We plan to obtain a repeat CT venogram within the next few months to guide further discussion regarding the need for chronic anticoagulation.

## Conclusions

This is a unique case of a patient with HZO, CST, and suspected HZM. Her management and hospital course were uncomplicated due to early intervention. Serial neurologic exams were crucial for the prompt detection of the rare but dangerous formation of CST.
